# Basics for the potential use of saliva to evaluate stress, inflammation, immune system, and redox homeostasis in pigs

**DOI:** 10.1186/s12917-022-03176-w

**Published:** 2022-02-28

**Authors:** J. J. Cerón, M. D. Contreras-Aguilar, D. Escribano, S. Martínez-Miró, M. J. López-Martínez, A. Ortín-Bustillo, L. Franco-Martínez, C. P. Rubio, A. Muñoz-Prieto, A. Tvarijonaviciute, M. López-Arjona, S. Martínez-Subiela, F. Tecles

**Affiliations:** 1 Interdisciplinary Laboratory of Clinical Analysis, Interlab-UMU, Regional Campus of International Excellence ‘Campus Mare Nostrum’, University of Murcia, 30100 Murcia, Spain; 2 Department of Animal Production, Regional Campus of International Excellence ‘Campus Mare Nostrum’, University of Murcia, Campus de Espinardo s/n, 30100 Espinardo, Murcia, Spain

**Keywords:** Biomarkers, Inflammation, Immune system, Oxidative status, Pigs, Saliva, Stress

## Abstract

The use of saliva as a biological sample has many advantages, being especially relevant in pigs where the blood collection is highly stressful and painful, both for the animal and the staff in charge of the sampling. Currently one of the main uses of saliva is for diagnosis and detection of infectious diseases, but the saliva can also be used to measure biomarkers that can provide information of stress, inflammation, immune response and redox homeostasis. This review will be focused on the analytes that can be used for such evaluations. Emphasis will be given in providing data of practical use about their physiological basis, how they can be measured, and their interpretation. In addition, some general rules regarding sampling and saliva storage are provided and the concept of sialochemistry will be addressed. There is still a need for more data and knowledge for most of these biomarkers to optimize their use, application, and interpretation. However, this review provides updated data to illustrate that besides the detection of pathogens in saliva, additional interesting applicative information regarding pigs´ welfare and health can be obtained from this fluid. Information that can potentially be applied to other animal species as well as to humans.

## Background

The use of saliva as a biological sample has many advantages, mainly related to its collection. It can be obtained by non-invasive and usually easy procedures, and the sampling does not produce pain. In addition, repeated specimens can be obtained anytime, anywhere, and without the need for specialized staff. Therefore, it is very suitable for monitoring purposes having many potential applications both in the veterinary and human field [1–4].

**Fig. 1:**
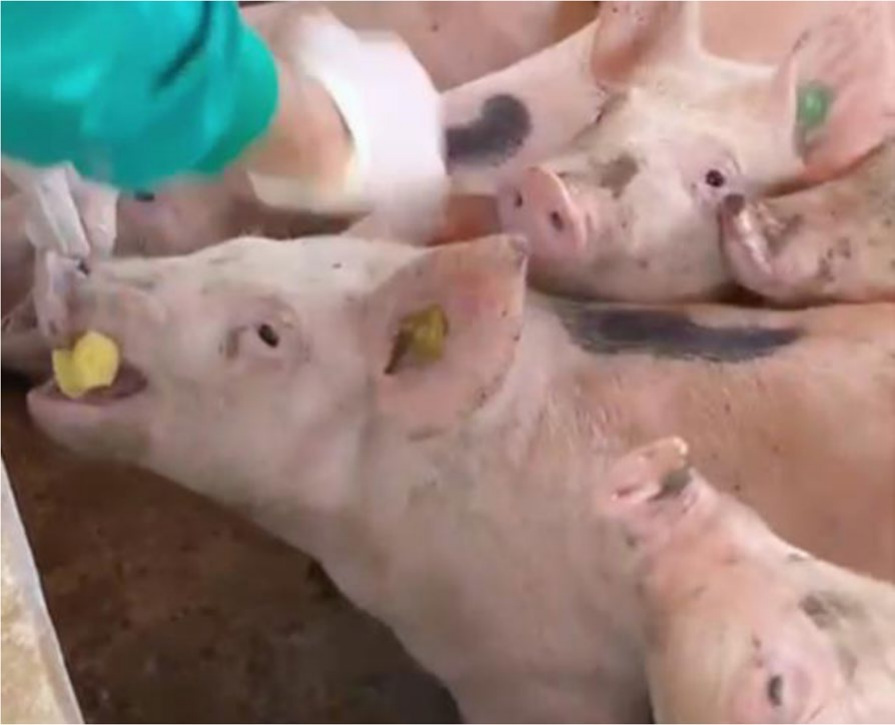
Saliva sampling in pigs

These advantages are especially relevant in pigs where the blood collection is highly stressful and painful, both for the animal and the staff in charge of the sampling [5, 6]. Therefore, the use of saliva in this species can be very appropriate on-farm and also in research. On farms, personnel can readily take the samples, leading to the possibility of more frequent analysis and better control of health and welfare (Fig. 1). This can allow faster and more focused interventions and therefore can produce a general improvement in quality and productivity [7]. In research projects on pigs, the saliva could substitute blood in some cases, such as measuring cortisol for stress evaluation [5, 8]. The no need for blood extraction in the experimental procedures will increase animal welfare, allowing better fulfilment of the Animal Research Care and Use Guidelines requirements.

**Fig. 2:**
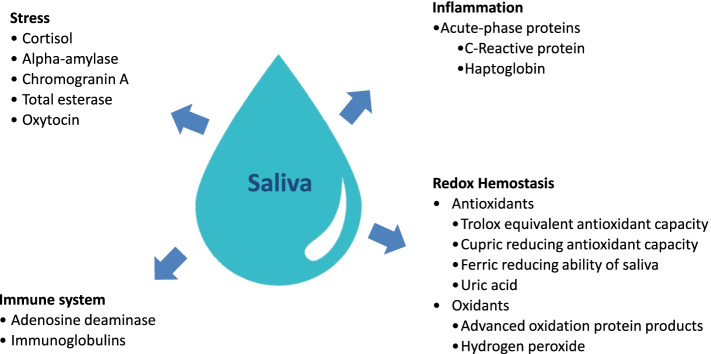
Analytes that can be measured in saliva to evaluate stress, inflammation, immune response and oxidative status

Looking more deeply into saliva applications, a comprehensive review of its use for the detection of pathogens in pigs has been recently published [9]. According to this publication, more than 23 viral pathogens can be detected in swine saliva, and currently the detection of infectious diseases is possibly the main use of saliva in routine practice in this species. However, the saliva can also be used to assess other aspects related to the pig health and welfare that can be of interest, such as the evaluation of stress, inflammation, immune response and redox homeostasis (Fig. 2). This review will be focused on these other aspects, giving information about the analytes that can be used for such evaluations. Emphasis will be given in providing data about their physiological basis, how they can be measured, and their interpretation in a concise and clear way that could be of practical use. In addition, a point about some general rules regarding sampling and saliva storage will be included, and the concept of sialochemistry will be addressed.

It is very important to point out that there is still a need for more data and knowledge for most of these biomarkers to optimize their use, application, and interpretation. The generation of this data in the future will help to better define the possibilities of saliva to evaluate stress, inflammation, immune system and redox status in pigs. However, it is expected that this review can help to extend the idea that besides the detection of pathogens, there is other interesting applicative information regarding pigs´ welfare and health that can be obtained from this fluid.

## Evaluation of stress

Detailed information about the main causes, consequences, and general biomarkers of stress in pigs can be found in a previous review [6]. In this point, we will focus on the biomarkers of saliva, and we will study: (1) cortisol that evaluates the hypothalamic-pituitary-adrenal axis (HPA), (2) salivary alpha-amylase (sAA) and chromogranin A (CgA) that are related to the autonomous nervous system (ANS), (3) total esterase (TEA) and some of their components such as salivary lipase (sLip) and butyrylcholinesterase (BChE) which are enzymes that have been related to situations of pain and discomfort. In addition, we will address the (4) oxytocin (OT), which is having a growing interest as a marker of positive emotions.

### Cortisol

#### Physiology and measurement

Currently, cortisol is possibly the most widely used biomarker to detect stress in pigs. When a stressful condition occurs, there is an activation of the HPA axis and the production of cortisol that is released into the blood. In blood, cortisol is present in two fractions: protein-bound cortisol and free cortisol; whereas in the saliva there is only the free cortisol which is the active fraction. The pass of free cortisol from blood to saliva is by passive diffusion of the molecule to the salivary gland [8].

Usually, cortisol in saliva is measured by methods based on antigen-antibody reaction, existing various formats such as radioimmunoassays, enzyme-linked-immunoassays (ELISAs) or automated chemiluminiscence immunoassays that have been validated in the saliva of pigs [10, 11]. In addition, cortisol in saliva can be measured by mass spectrometry [12].

#### Interpretation

There is a high number of published reports published in which cortisol has been measured in the saliva of pigs. Although their detailed description is beyond the objective of this review, some general ideas can be obtained from them that could help in the interpretation of this analyte:

1. *There is variability in the references ranges reported in the literature for cortisol in non-stressed animals and a high intraindividual variation.* The mean values for cortisol reported in saliva in adult animals can vary depending on the authors, as can be observed in Table 1. This could be due to the different types of assays used, and intrinsic factors such as breed or age. It is also important to indicate that cortisol concentrations follow a circadian rhythm in saliva, that can vary with age and sex [13]. The variability between animals can reach a coefficient of variation of cortisol of 62% in the saliva of non-stressful subjects [5].

**Table 1:** Some examples of reported values for salivary cortisol in healthy pigs

Animals	Breed	Sample size	Method	Cortisol mean values (μg/L)	Commentary	Reference
Finishing pigs (165 days old, ~ 100 kg)	Duroc x [Landrace x LargeWhite]	20	Immuno-chemiluminescence	2.4–7.0		Escribano et al. [10]
Growing pigs (~ 105 days old, ~ 88 ± 8 kg)	• Duroc × [Landrace × Large White] • L62 (a crossed of several genotypes) × [Landrace × Large White] • Pietrain × [Landrace × Large White]	12 samples from ropes (10–12 animals per rope)	Liquid chromatography–tandem mass spectrometry	• 1.20 ± 0.20 • 0.51 ± 0.05 • 0.74 ± 0.30	No individual samples were measured, but 12 rope samples from 4 different farms (3 ropes per farm)	Rey-Salgueiro et al. [12]
Nulliparous non-pregnant gilts (8–9 months old, ~ 150–170 kg)	Large White × Landrace	91	Radio immunoassay	0.8–2.2	Samples correspond to 8 gilts repeatedly sampled at different days	Merlot et al. [5]

Based on the data indicated above, it would be recommended to compare the cortisol concentrations in an individual with the values obtained (a) with the same assay and ideally, (b) in the same animal or group of animals with similar age, breed and sex conditions and without evident signs of stress.

-2. *Diverse acute stimuli can produce different increases in salivary cortisol.* For example, in a report in which the response of salivary cortisol to different potential acute stressful situations was evaluated, the highest increases in cortisol were obtained for snaring, relocation and vena cava blood sampling. In these situations, cortisol showed the highest values at 15–30 min after the stimulus, whereas the response to a meal or tail blood sampling did not elicit significant increases in this analyte [5] (Table 2).

**Table 2:** Some examples of cortisol (saliva and plasma) responses after different stressful stimuli [5]

Animals	Stressful condition	Sample size	Sampling times	Cortisol values (μg/L): (Before stress vs peak after stress)	Commentary
Nulliparous non-pregnant gilts (8–9 months of age, ~ 150–170 kg)	Vena cava blood sampling	8	Before and 15, 30, 60, 90 and 120 min after the stressful condition	• Saliva (1.7 vs 3.2) • Plasma (10.5 vs 35.8)	• In saliva peaked at 15 min • In plasma peaked at 15 min, still increased at 30 min (no significant changes at other times)
	Snaring	8		• Saliva (0.8 vs 5.1) • Plasma (11.8 vs 47.4)	• In saliva peaked at 15 min, still increased at 30 min • In plasma peaked at 15 min, still increased at 60 min (no significant changes at other times)
	Relocation	8		• Saliva (1.5 vs 3.3) • Plasma (8.9 vs 35.0)	• In saliva peaked at 15 min, still increased at 30 and 60 min • In plasma peaked at 15 min, and increased until 120 min

-3. *The salivary cortisol variation in chronic stress should be evaluated more deeply.* There is controversy at  this point since although some reports indicated no significant increases in situations of chronic stress [14], other studies suggest that salivary cortisol could be a possible marker of chronic stress in pigs [15, 16] as occurs with the cortisol in hair [17]. Therefore, further studies to elucidate the role of salivary cortisol in chronic stress should be performed.
-4. *In addition to cortisol, other steroids such as corticosterone or testosterone can be measured in the saliva of pigs.* In a report, cortisol in saliva determinations was more sensitive and their results less variable than corticosterone to detect stress [12]. Regarding testosterone, increases in saliva of pigs in different stressful situations have been described [18], and interestingly increases in testosterone with cortisol values in reference range have been associated with predisposition to aggressive behavior in humans [19].

### Salivary alpha-amylase (sAA) and chromogranin-a (CgA)

#### Physiology and measurement 

Both biomarkers are directly produced by the stimulation of the salivary glands by the ANS.sAA can be directly quantified by immunoassays, or it can be evaluated by its enzymatic activity by spectrophotometric assays, being the last one more sensitive to detect stressful conditions in pigs [20] and humans [21]. CgA can be measured by immunoassays in the saliva of pigs [22]. The assays used for the measurements of sAA and/or CgA are easier to perform and cheaper than the analysis of adrenalin and nor-adrenalin, which are the classical analytes used to evaluate the sympathetic activity.

#### Interpretation

Some general ideas can help in the understanding of these analytes:

1. *CgA and sAA can increase in situations of acute stress (*Table 3*).* After acute stress induced by a snaring, sAA and CgA showed variability in their response and even did not increase in some pigs [20, 22, 23]. This situation also happens with cortisol [22], and although it is not known the cause, it reflects a variability in the individual response to this model of acute stress. In this line, sAA can increase from values lower than 100 IU/L to higher than 1000 IU/L, with some individuals reaching up to 4000 IU/L, at the moment of the stress induction by snaring in some pigs, whereas it does not change in other pigs [20]. CgA was reported to increase in a snaring around 1.2-fold just after 10 min of continuous restraining [24] and from values lower than 2 mg/L to up to 3 mg/L after 15 min of ceasing the snaring [22]. In a model of restraining by enclosure in a steel cage for 60 min, the increase in CgA was higher than 4-fold and persisted increased 2–3-fold at 30 min after ceasing the stimulus [24]. This could indicate a possible relation of this marker with stress of longer duration as will be discussed later.

**Table 3:** Some examples of salivary chromogranin A (CgA) and alpha-amylase activity (sAA) response after different stressful stimuli

Stressful condition	Reference	Animals	Sample size	Sampling times	Biomarker	Results (unless indicated, median values are expressed)	Comment
<5 min snaring	Contreras-Aguilar et al. [20]	Mid-fattening period (104.8 ± 10.0 days, 78.3 ± 6.3 Kg) male Duroc x [Landrace x LargeWhite]	6	Before, during stress and 15 min after stress	sAA (IU/L)	It reaches values from < 100 to > 1000 during stress, but without statistically significant differences	High interindividual variability, with increases observed in 4 out of 6 animals
	Fuentes et al. [23]	Mid-finishing fattening period (90–120 days	12	Before, just after, 30 and 60 min after stress	sAA (IU/L)	Significant increases from ~ 200 to > 1000 at 30 and 60 min	High interindividual variability, with increases observed in 8 out of 12 animals
	Escribano et al. [22]	Early-fattening period Duroc x [Landrace x LargeWhite]	15	Before, 15 and 30 min after stress	CgA (μg/mL)	Increases observed from 1.75 to 2.56 μg/mL at 15 min	Increases observed in 13 out of 15 animals
10 min snaring	Huang et al. [24]	Male growing pigs (Large White × Landrace × Duroc)	10	Before, during and 30 min after stress	sAA (IU/L)	From ~ 450 to ~ 500 at 30 min, without statistically significant changes	Previous 2 h food deprivation could lead to high basal values
					CgA (protein expression)	Increased significantly, peaking at 10 min during stress (1.2-fold increase); remained high at 30 min after stress	
60 min enclosure in a steel cage	Huang et al. [24]	Male growing pigs (Large White × Landrace × Duroc)	10	Before, during and 30 min after the stress	sAA (IU/L)	Values ranged from ~ 400 to ~ 450, without statistically significant changes	
					CgA (protein expression)	Increased significantly, peaking at 60 min during stress (> 4.0-fold); remained high 30 min after stress	
Weaning	Escribano et al. [25]	Piglets (25 days old)	102 (51 female and 51 male)	1 day pre-weaning, and 1 and 2 days post-weaning	sAA (IU/L)	Values ranged from ~ 12,000 to ~ 15,000 postweaning, without statistically significant changes	High interindividual variability.
					CgA (μg/mL)	Increase from 0.64 ± 0.8 to 4.01 ± 5 μg/mL 1 day post-weaning	Still increased 2 days post-weaning with mean values of 3.11 ± 3

In some cases, there can be divergences between sAA and CgA. For example at weaning, which is a known stressful condition, there were increases in salivary CgA, which were correlated with skin lesions, but sAA did not show changes [25]. Although no data exist regarding sAA, CgA in saliva of pigs seems to be not affected by circadian rhythms [26].

-2. *The behavior of CgA and sAA in saliva in chronic stress should be evaluated more deeply.* Although both biomarkers are related to the ANS, and therefore to the reaction occurring after acute stress, changes in CgA and sAA have been described in situations of stress of longer duration, for example:
  sAA showed increases in pigs suffering  pain due to lameness and rectal prolapse [27]. The animals with these disorders showed even higher increases in sAA  (7.49-fold and 18.20-fold in most severe cases of lame and prolapsed animals, respectively, compared to healthy animals) than in other biomarkers such as cortisol (1.72-fold and 2.30-fold in lame and prolapsed animals, respectively). Maybe the longer duration of the pain could have influenced the higher values of sAA [27].
  Although the mechanism is not well known, CgA decreased in saliva after different types of environmental enrichment and herbal supplementation during 2 months in growing pigs, from values of 1 mg/L to values lower than 0.3 mg/L. This could indicate a reduction in the stress of these pigs during this period. In addition, CgA in saliva showed a low to moderate, but significant, correlation with cortisol concentration in hair [28].
-3. *CgA is an early biomarker of postpartum dysgalactia syndrome (PDS).* CgA was significantly increased in sows with PDS before farrowing, showing a higher sensitivity than other markers such as cortisol. Although the reason for this increase is unknown, the high values of CgA could  indicate a situation of stress with activation of the adrenergic system that could be involved in the pathogenesis of this disease. In addition, there could be a role of CgA in the gastrointestinal disorders associated with PDS, since in the human gastrointestinal tract, CgA is released from enterochromaffin cells and neurons of the submucosal and myenteric ganglia, and may modulate colonic motility in response to inflammation [29].

### Total esterase activity (TEA) and its components

#### Physiology and measurement

TEA is abundant in the saliva of pigs [30]. Several enzymes contribute to this esterase activity:

- Cholinesterase (ChE) and cholesterol esterase represent around 20% of salivary TEA activity in healthy non-stressed pigs [30]. Whereas acetylcholinesterase is the predominant form in human saliva [31], butyrylcholinesterase (BChE) is the predominant isoenzyme in porcine saliva [32]. The origin and function of ChE in saliva remain unknown, but the lack of correlation between serum and salivary ChE [33] supports the idea that the enzyme could be secreted by the salivary gland. In addition, ANS activity could be implied in ChE production [34].
- Salivary lipase (sLip) could be around 30% in healthy non-stressed pigs. Although its main function is related to triglyceride digestion, lipase secretion by salivary glands could also be related to the activation of the sympathetic nervous system [35].
- Carbonic anhydrase isoenzyme 6 (CA-VI) could represent up to 50% of the TEA activity in porcine saliva [30]. In humans, this enzyme (also called gustin) is related to taste function and taste bud growth [36]. Despite this, its secretion is also related to the sympathetic nervous system [37].

TEA, BChE and sLip can be measured spectrophotometrically, and there are assays validated for pigs [30, 32].

#### Interpretation

1. *Salivary TEA can increase in situations of acute stress.* Increased salivary TEA activity in pigs, with an increase of 1.49-fold just after the stimulus, has been described after a restraining by nasal snare. In addition, an increase (1.8-fold) in salivary TEA was reported at 4 h after transport and lairage at slaughterhouse compared with values before transport. This increase was lower than those observed with other biomarkers such as cortisol [38].
2. *The behavior of TEA in saliva in chronic stress should be evaluated more deeply.* Salivary TEA could also be increased in pigs suffering pain due to lameness and rectal prolapse [27]. Therefore, further studies would be of interest to evaluate the behavior of TEA in situations of pain or stress of long duration.
3. *The change of the different components of salivary TEA can provide additional information.* Measuring some components of TEA such as BChE and sLip could provide additional information since the changes observed in those enzymes can be different depending on the stressful stimulus. For example, in case of acute stress such as a nasal restraining or transport and lairage at slaughterhouse during 4 h, BChE could be increased more than 5-fold compared with the pre-stress values [32], being more sensitive than TEA (which increased less than 2-fold) [30] (Table 4).

**Table 4:** Some examples of salivary total esterase activity (TEA), butyrylcholinesterase (BChE) and lipase response after different stressful stimuli

Stressful condition	Reference	Animals	Sample size	Sampling times		Results (UI/L, unless indicated, median values are expressed)	Comment
Transport and lairage to slaughterhouse	López-Arjona et al. [38]	Female at end-fattening period (5–6 months) LargeWhite x Pietrain	45	Before, immediately after and at 4 h from transport	TEA (IU/L)	• Before transport:151.7 • Just after: 190.6 • After 4 h: 277.8	
Disease (lameness and rectal prolapse)	Contreras-Aguilar et al. [27]	Male mid fattening period (60–90 days) (Large White)	60	Just only one sampling	TEA (IU/L)	• Healthy: 128.8 • Lame: 293.2 • Prolapsed: 780	
2 min snaring	Tecles et al. [30]	Male growing pigs (Large White × Landrace × Duroc)	20	Before, just after and at 15 min after the stress	TEA	1.49-fold increase just after stress	Returned to basal values at 15 min
					Lipase	1.60-fold increase at 15 min after stress	No increase just after stress
	Tecles et al. [32]				BChE (nmol/min/mL)	5.25-fold increase just after stress	Still increased at 15 min

### Oxytocin (OT)

#### Physiology and measurement

OT is a hormone that, in addition to its physiological role in labour and lactation, is considered a biomarker of positive emotions and social well-being in domestic animals. The increase of OT with positive welfare situations is in contrast with the rest of the biomarkers of stress used until now, which increase when there is a stressful situation or negative welfare [39]. The source of OT in the saliva is unknown, but a recent report in humans indicates that salivary OT can reflect endogenous concentration and production. In this paper, repeated intranasal administration of OT induced long-lasting changes in endogenous salivary OT levels, presumably through a positive spiral of OT release [40].

In the saliva of pigs, as in other species, OT can be in two main forms: linked to proteins or free. In addition, different OT metabolites can exist [41, 42]. Usually, immunoassays are used to measure OT in saliva, and recently two assays, which do not need extraction or lyophilization, were specifically validated for pigs’ saliva . One seemed to have more affinity for detecting the OT linked to proteins, whereas the second one ould detect other forms [39]. In addition to these immunoassays, other assays  that usually need reduction and alkylation [42] or extraction [43] have been applied in pigs. Although there are no studies in pigs, high-performance liquid chromatography-mass spectrometry (HPLC–MS) can also be used for detection of OT in saliva, giving in general lower values of OT concentrations than immunoassays [44].

#### Interpretation

As other biomarkers, the knowledge about OT measurements in the saliva of pigs is in its beginning, but there is some information that can be of interest and application (Table 5):

**Table 5:** Some examples of oxytocin (OT) response after different stimuli

Stressful condition	Reference	Animals	Sample size	Sampling times	Results (UI/L, unless indicated, median values are expressed)	Comment
Transport and lairage to slaughterhouse	López-Arjona et al. [38]	Female at end-fattening period (5–6 months) LargeWhite x Pietrain	45	Before, immediately after and at 4 h from transport	Between 1.38–1.73-fold decrease at 4 h, depending on the method	Two methods used (one with a monoclonal and other with a polyclonal antibody)
Positive human-animal interaction	Lürzel et al. [43]	LargeWhite × Landrace sows (22 ± 7 months old and 48 days pregnant)	18	Just before and after the trial	No significant changes were observed	Positive correlation between OT concentration and duration of being stroked
Ejaculation	López-Arjona et al. [45]	Boars of three different breeds (2 Landrace, 13 Duroc, 18 Pietrain)	33	24 h before ejaculate collection, immediately after starting the ejaculation and 2 h after ejaculation	Significant increase from 775.6 (24 h before) to 1077.0 pg/mL (at starting ejaculation)	Higher responses in younger boars, boars with higher libido intensity and Pietrain breed

1. *Different assays can measure diverse OT forms or metabolites and give different values.* For example, in the report where two different assays were validated in the saliva of pigs, one gave values in μg/L, whereas the other gave values in ng/L of OT [39]. In addition, it should be pointed out the intraindividual variability of OT values, since in one report in a group of 45 adult pigs the 25th and 75th percentiles values could reach to 2-fold the mean values [38]. More details about the ability of different assays to give diverse values can be found in a recent review [41].
2. *Some physiological conditions such as farrowing and lactation can produce changes in OT in saliva.* OT concentrations were significantly higher at the beginning of lactation, and also these changes were differently detected depending on the assay used. In this case, a commercially available assay was less sensitive to detect these changes [42].
3. *OT in saliva can decrease in situations of acute stress.* In a report, decreases in salivary OT concentrations were found in pigs at 4 h of lairage at a slaughterhouse. This could indicate a reduction of positive feelings in this situation, possibly due to various stressful stimuli such as the unloading process, mixing with unfamiliar pigs and stranger sounds. Depending on the assay used, these decreases were of different magnitude (42.2% versus 27.5%), so also the type of assay can influence the detection of changes after stressful situations [38].
4. *OT can increase by positive interactions.* In a study in which positive human-animal interactions were evaluated in pigs, the concentration of OT in saliva was associated positively with being stroked [43]. In addition, ejaculation increases salivary OT concentrations in breeding boars [45]. Therefore, this data suggest that this hormone can be a marker of positive emotions.

## Evaluation of inflammation

### Acute phase proteins (APPs)

#### Physiology and measurement

APPs are proteins that change in concentrations in response to inflammation, being considered the most sensitive and early biomarkers for this process. Recently, based on the knowledge about physiology and clinical application of these proteins, an updated seven-point plan for the APPs use and interpretation in veterinary medicine was reported, that can also be applied to saliva in pigs [7].

**Fig. 3:**
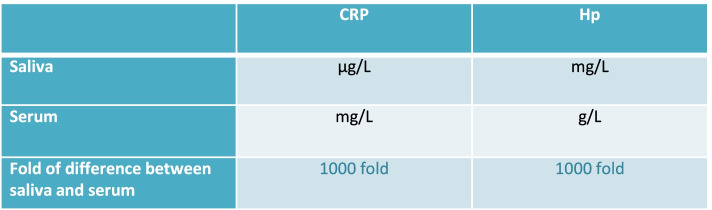
Units of measurement of CRP and Hp in saliva and serum and fold change [7]

The most important particularity regarding APPs measurements in saliva is the fact that concentrations of most important APPs such as C-reactive protein (CRP) or Haptoglobin (Hp) are at approximately 1000-fold lower concentrations in saliva than in serum. For example, in the case of CRP the concentration in serum is in mg/L, whereas in saliva is μg/L. A similar fact happens for Hp, being in serum in g/L but in the saliva in mg/L [7] (Fig. 3). This would be the reason why some attempts to measure APPs in the saliva with conventional assays can have no success. Therefore, the use of more sensitive assays to detect APPs in the saliva are recommended. For example, time-resolved fluorometric or alphalisas assays have been successfully used to measure APPs in the saliva of pigs and other species as humans [46, 47]. Alphalisa assays have as their main advantages the use of a lower sample volume and the no need for washing steps [47].

The mechanisms which are responsible for the presence of APPs in saliva are still to be elucidated. However, in pigs as in other species such as dogs or humans, some APPs like CRP were significantly correlated in saliva and serum; and recently a transport process for CRP from blood to saliva in humans has been described [48].

#### Interpretation

As a general idea, the rules for interpretation of APPs in saliva in pigs will be similar to those reported for other species [7]. However, some particularities can be indicated for saliva and can help in the interpretation of the results obtained:

1. *Although baseline values are lower than in serum, the distinction between major and moderate APPs can also be made in saliva.* Hp values in the saliva of healthy adult pigs are usually lower than 1 mg/L and can reach to 3–4 mg/L in pigs with inflammation, whereas CRP in healthy animals usually has lower values than 10 μg/L and can reach values up to 100 μg/L in inflammation [49] (Table 6). This could indicate that, as reported in serum [52], Hp is a moderate acute phase protein, whereas CRP is a major APPs in saliva of pigs. This could also explain that in the case of some more chronic inflammatory states, salivary Hp might be a more sensitive marker than CRP [49], possibly due to the moderate nature of Hp that maintain its increases during longer time.

**Table 6:** Some examples of acute phase protein (CRP: C-reactive protein; Hp: haptoglobin) response after different stimuli

Stressful condition	Reference	Animals	Sample size	Sampling times	Biomarker	Results (unless indicated, median values are expressed)	Comment
Three lipopolysacharide (LPS) injections at 48 h intervals	Escribano et al. [49]	Pietrain × (Landrace × LargeWhite) growing pigs (77 days old)	10	3 days before and 3 h after each LPS administration	CRP (μg/L)	From < 10 to > 60 after 1st and 2nd injections	No increased after 3rd injection
					Hp (mg/L)	From < 0.5 to > 3 after 1st injection	Still increased after 2nd and 3rd injections
Feed deprivation	Ott et al. [50]	Pietrain Plus × Rattlerow Seghers growing pigs	24	24 h before, just after refeeding and 3 days after	Hp (mg/L)	Significantly increased after refeeding (~ 0.75) compared with 3 days later (< 0.5)	
5 days of isolation and later regrouping	Escribano et al. [51]	Duroc × (Landrace × Large White) males (190 days old)	7	Once a day for pre-stress period (5 days), isolation (5 days) and regrouping (3 days)	CRP and Hp	No significant differences observed with baseline or with control group (*n* = 7)

-2. *APPs are highly sensitive to detect inflammation, but they have a low specificity to detect the cause of inflammation.* The APPs can detect inflammation with high sensitivity and very early, in many cases before the appearance of clinical signs, being an excellent marker of the presence of subclinical diseases. However, they usually do not provide information about the cause of inflammation; therefore, it should be combined with other tests or clinical data. For example, if an infectious disease is suspected, the detection of the pathogen, which could also be made in saliva [9], would be recommended.

In line with this low specificity, increases in APPs in saliva have been found not only in inflammation but also in stress situations. However, the relation between APPs and stress is controversial. For example, Hp in saliva increased in stress induced by feed deprivation [50] and a link between APPs and stress has been hypothesized [53]. However, in other stress situations no changes in APPs have been found. For example, pigs exposed to a psychosocial stressor, that increased their cortisol levels, did not increase the Hp and CRP concentrations in saliva [51].

## Evaluation of immune system

### Adenosine deaminase (ADA)

#### Physiology and measurement

ADA is a ubiquitously expressed enzyme that appears in saliva. It has two isoenzymes: ADA1 and ADA2, which have a role in the differentiation of T lymphocytes. Total ADA (tADA) activity represents the sum of the activity of the two isoenzymes, and in humans its measurement in serum has been used as a biomarker of cell-mediated immunity and chronic inflammation [54, 55]. There is still a lack of knowledge about the origin and sources of ADA in saliva, being the clarification of this aspect of high interest, since for example tADA in pigs is over 100-fold higher than in serum [56, 57].

The activities of tADA and its isoenzymes can be measured in saliva by a three-step procedure [57]: (1) the sample is measured for evaluation of tADA by a spectrophotometric assay; (2) the sample is measured again with the same assay adding Erythro-9-2-hydroxy-3-nonyl adenine (EHNA), which is an inhibitor of ADA1, at the appropriate concentration for ADA2 estimation; and (3) the isoenzyme ADA1 is calculated by the difference between measurements of step 1 and 2.

#### Interpretation

Although the knowledge about ADA is still in its infancy, currently some data can be given that could be used for its interpretation (Table 7):

**Table 7:** Some examples of immunity salivary biomarkers (ADA: adenosine deaminase; Ig: immunoglobulin) response after different stimuli

Stressful condition	Reference	Animals	Sample size	Sampling	Biomarker	Results (unless indicated, median values are expressed)	Comment
Lameness	Contreras-Aguilar et al. [57]	LargeWhite x LargeWhite males (2–3 months-old	20	10 pigs showing lameness were compared with 10 healthy animals	ADA (IU/L)	Significantly increased in lame pigs (1829.0) compared with control (495.1)	Higher sensitivity than the measurement of ADA in serum
Lameness	Contreras-Aguilar et al. [27]	LargeWhite male growing pigs (2–3 months old)	66	• Healthy (*n* = 22) • Lame (*n* = 21) • Rectal prolapse (*n* = 23)	ADA (IU/L)	• 620.5 • 1108.3 (non-severe) to 1785.6 (severe) • 593.7 (non-severe) to 3171.9 (severe)	Only those severe cases showed significant changes when compared with healthy
PRRS and PMWS	Escribano et al. [58]	Duroc × (Landrace × Large White) males (~ 120 days old)	30	Healthy (n = 15) vs Diseased (*n* = 15)	IgA (mg/L)	101.4 vs 234.0	IgG showed the largest difference, probably due to the humoral immune response
					IgG (mg/L)	9.09 vs 102.6	
					IgM (mg/L)	16.3 vs 65.3	
Threee lipopolysacharide (LPS) injections at 48 h intervals	Escribano et al. [49]	Pietrain × (Landrace × LargeWhite) growing pigs (77 days old)	10	3 days before and 3 h after each LPS administration	IgA	From < 100, peaked > 400 after 2nd injections	Increased also after 1st and 3rd injections
Restrain with iron Fences for 20 min	Muneta et al. [59]	LargeWhite male piglets (6–8 weeks old)	8	After 5 min, at 10 and 20 min after starting stress, and 10 min after ceasing stress	IgA	~ 500% increase at 10 and 20 min after starting stress	Returned to baseline 10 min after ceasing stress

1. *ADA is present in high amounts in the saliva of pig.* Compared with other species such as dogs, horses, or cows, pigs showed the highest tADA values in saliva [57].
2. *ADA increases in saliva in inflammatory diseases.* In saliva, significant increases have been reported  in tADA and ADA1 in pigs with lameness compared with healthy pigs. This study showed a high correlation between tADA and its isoenzymes with serum CRP, indicating that salivary ADA activities are related to inflammation [57]. It is important to point out that, in this study, saliva was more sensitive than serum to detect these changes, being this situation an example in which saliva would be preferred to serum in the measurement of an analyte. Although this should be further explored, ADA could also have potential in pigs as a tool for pain assessment in inflammatory situations such as lameness and rectal prolapse [27].

### Immunoglobulins (Igs)

#### Physiology and measurement

In saliva there are IgG, IgM and IgA. Salivary IgG and IgM are mainly derived from blood, whereas IgA is mainly produced by the salivary glands in plasma cells. IgA concentration seems to be more influenced by stress, whereas IgG and IgM in saliva would be more related to its concentration in serum. Also, it is interesting to indicate that IgM is produced earlier than IgG in disease and both are markers of the humoral acquired immunity, being IgM also related to the innate immune system [59–61]. The three different types of Igs can be measured in the saliva of pigs by immunoassays with adequate analytical performance [58] (Table 7).

#### Interpretation

1. *The Igs types are at different concentrations in saliva.* IgA is in a higher amount in saliva in healthy animals than IgG and IgM; having healthy pigs concentrations of IgA in the range of 100 mg/L, whereas IgG are in 10 mg/L and IgM in 20 mg/L [58].
2. *IgG shows major increases in situations of infectious diseases.* Specific IgGs are more used for detection of antibody production against an infectious agent [9]. However, the measurement of their total values can also have clinical value. For example, IgG seems more sensitive than other Igs to differentiate between healthy and diseased pigs with porcine reproductive and respiratory syndrome (PRRS) and post-weaning multisystemic wasting syndrome (PMWS), with a difference of more than 10-fold between groups [58]. Therefore, an increase in IgG in saliva could raise the suspicion of an infectious disease.
3. *IgA can increase in cases of inflammation but also after stressful conditions.* Regarding the relation with inflammation, an increase in IgA in saliva was reported associated with endotoxemia with increases from 50 mg/L in basal levels to a peak of mean values of 400 mg/L that can even reach 500 mg/L. In addition, it has been postulated that IgA could also be a marker of more chronic inflammatory states [49]. On the other hand, saliva IgAs can increase after different stressful conditions in pigs such as after a restrain stress; from basal levels around 100 mg/L to a peak of mean values around 500 mg/L that can even reach 800 mg/L. These increases were higher than those of cortisol and returned to pre-stress levels sooner after removing of the stress [59]. In this line, it has been proposed in humans that salivary secretion of IgA might be linked to the activation of the ANS system [62].

## Evaluation of the redox homeostasis

### Physiology and measurement

To our knowledge, currently in saliva of pigs it can be measured the total antioxidant capacity (TAC) by three assays: Trolox equivalent antioxidant capacity (TEAC), cupric reducing antioxidant capacity (CUPRAC), and ferric reducing ability of saliva (FRAS, which has the same basis that ferric reducing ability of plasma or FRAP). Moreover, other antioxidants such as uric acid and catalase can be measured. In addition, oxidant biomarkers such as advanced oxidation protein products (AOPP) and hydrogen peroxide (H_2_O_2_) can be quantified in porcine saliva [63, 64]. The origin of these analytes in saliva is unclear, and only CUPRAC showed a significant positive correlation between saliva and plasma values, being this correlation low [63].

All these biomarkers can be measured by spectrophotometric assays and adapted to automated analyzers [63].

### Interpretation

At this time, with the current knowledge we could provide the following ideas about the interpretation of the markers of redox homeostasis:

1. *The values of biomarkers of redox homeostasis in saliva change in some physiological conditions such as farrowing and lactation.* Although reference ranges should be established with larger populations and the values could change depending on the assays used and therefore these values should be interpreted with caution; in our experience, the mean values that we usually obtain in the saliva of healthy adult animals are between 0.1–0.5 mmol/L for TEAC, CUPRAC and FRAS, 30–200 μmol/L for AOPP and 5–25 μmol/L for H_2_O_2_ [27, 63, 64].

There is an increase in antioxidant and oxidant concentrations in the saliva of sows at the first day of lactation, of around 1.2–2 fold, which decreased during the 20 days of lactation, in line with the previously described increases of various biomarkers of oxidative status in serum of sows during early lactation [63].

-2. *Changes in biomarkers of redox homeostasis occur in different situations in which there are disturbances in the pig.* For example, piglets supplemented with high doses of garlic exhibited decreased antioxidants biomarkers, such as CUPRAC, and increases in oxidant biomarkers such as H_2_O_2_, that could reflect the oxidative effects described in farm animals after the consuming garlic at high doses [64].

In addition, pigs with prolapses and pain showed higher levels of FRAS, AOPP and H_2_O_2_ in saliva compared with the healthy animals, being salivary FRAS and AOPP correlated with the pain of the animals [27].

-3. *Interpreting the paradox.* On some occasions, a paradox can occur in which increases in antioxidant and oxidants are detected at the same time. This could reflect a situation in which an increase in antioxidants is produced to compensate the overproduction of oxidant compounds that is occurring. This means that, in some cases of oxidative disturbance, the balance would be tried to be reestablished.
-4. *It would be recommended in the future to evaluate the salivary biomarkers of redox homeostasis change in selected diseases and establish possible profiles for their evaluation.* If possible, these evaluations should include panels with at least two antioxidant and two oxidant biomarkers*.* These studies will open new possibilities of using saliva as a non-invasive sample to evaluate oxidative stress in pigs.

## General recommendations for saliva sampling and management

**Fig. 4:**
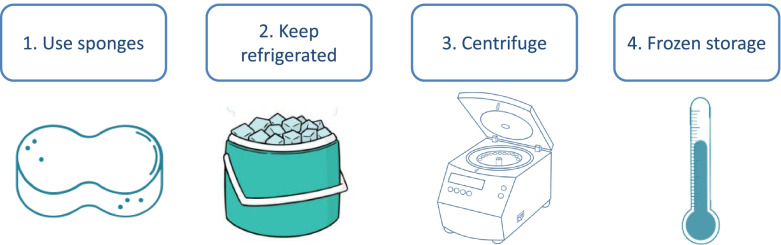
Recommendations for saliva sampling and management

We could make the following general recommendations for saliva sampling and management in pigs (Fig. 4):

1. The use of sponges. This allows to obtain stimulated saliva with less mucin content and therefore reduced viscosity leading to easier processing and management of the samples.
2. If samples are not processing in a short time, refrigeration or a least keeping them in a cool place is recommended, until their arrival to the laboratory.
3. Samples should be centrifuged to remove cell and food debris. The protocol of centrifugation that we use at our laboratory is 3.000×g for 10 min at 4 °C.
4. Ideally, samples should be stored at − 20 °C or − 80 °C if they will not be analyzed immediately. The optimal temperature for storage depends of the analyte. For example, CgA and ADA are stable in saliva 1 year stored at − 20 °C; whereas − 80 °C is recommended for prolonged storage of sAA or BChE and the enzymes and biomarkers of oxidative stress in general [26, 65]. Overall, at − 80 °C, the analytes are more stable than at − 20 °C. Information about how analytes in saliva can be stable at different storage conditions has been reported in pigs [65] and humans [66].

## Future perspectives: the concept of sialochemistry

In addition to the analytes described here, it is gaining attention the concept of ***sialochemistry,*** which is the application in saliva of the same or similar analytical profiles that are used in routine for the clinical biochemistry of serum.

For example, a profile of a total of 11 analytes including aspartate aminotransferase (AST), alkaline phosphatase (ALP), γ-glutamine transferase (GGT), lactate dehydrogenase (LDH), creatine kinase (CK), urea, creatinine, triglycerides, lactate, calcium and phosphorus was validated from the analytical point of view in the saliva of pigs with satisfactory results [67]. One advantage of this sialochemistry profile is the fact that most of the assays included are measured by spectrophotometric methods and can be easily set up in saliva with the commercially available reagents that are usually employed in clinical chemistry laboratories, being easily adapted to automated analyzers, manual or automated spectrophotometers or plate readers.

In addition, changes in this profile were studied in gestation, farrowing and lactation. Increases in muscle and hepatic enzymes (CK, AST, ALP, GGT and LDH) were detected at farrowing, and triglycerides increased at the end of gestation and remained at high concentrations until the end of lactation [46]. In particular, LDH is also increased in situations of reduced welfare in pigs [68]. In other species such as dogs, increases in urea and creatinine in saliva have been described in cases of renal failure [69] and increases in CK in muscle damage [67].

There is still much knowledge to be generated for the basis of the interpretation of the analytes that can be included in the sialochemistry profile in saliva. To help in this interpretation, the clarification of the mechanism involved in the presence of each analyte in saliva, and data about their correlations between serum and saliva, will be especially useful. In this regard, it should be pointed out that there are analytes present in saliva that are produced locally in the salivary glands. In addition,  the analytes can pass from blood to saliva by different ways such as: passive diffusion in the case of lipophilic molecules like steroid hormones, active transport in case of some proteins or filtration through spaces between acinus and ductal cells in case of small molecules [4].

Possibly some of these analytes could have a similar interpretation and clinical value that in serum, such as it was reported for urea and creatinine in dogs [69] and also occurs with the acute phase proteins; whereas others could have a different interpretation. In addition, some other analytes could be more sensitive to detect some processes when measured in saliva than in serum, as occurs with ADA  [57]). Ideally, specificity and sensitivity of different analytes in saliva in comparison with serum or urine should be evaluated in different stress or specific disease conditions in pigs, as it has been made in humans [70]. This evaluation could be done by individual analytes or also combined in algorithms.

## Conclusions

The saliva of pigs can be used to measure biomarkers that can help to evaluate stress, inflammation, immune system and redox homeostasis. These biomarkers, as well as the sialochemistry profiles, reflect that the saliva, in addition to be a diagnostic tool for infectious disease detection, can provide potential interesting information about the health and welfare status of the pig. However, there is still need of more data in order to validate the use of saliva in this field. Therefore, it is expected that in the near future, more knowledge about their physiology and practical applications will be generated on the salivary markers reviewed here, and also on new ones that could be discovered, especially by “omics” techniques. This knowledge expected to be generated will provide more precise and useful information about these biomarkers, contributing to a wider use of saliva in this species as well as other animal species and humans in the future.

## Data Availability

All data analyzed during this study is included in this published article.

## Abbreviations

ADA: Adenosine deaminase
ADA1: Adenosine deaminase isoenzyme 1
ADA2: Adenosine deaminase isoenzyme 2
ALP: Alkaline phosphatase
ANS: Autonomous nervous system
AOPP: Advanced oxidation protein products
APPs: Acute phase proteins
AST: Aspartate aminotransferase
BChE: Butyrylcholinesterase
CA-VI: Carbonic anhydrase isoenzyme 6
CgA: Chromogranin A
ChE: Cholinesterase
CK: Creatine kinase
CRP: C-reactive protein
CUPRAC: Cupric reducing antioxidant capacity
EHNA: Erythro-9-2-hydroxy-3-nonyl adenine
ELISA: Enzyme-linked-immunoassay
FRAS: Ferric reducing ability of saliva
Hp: Haptoglobin
H_2_O_2_: Hydrogen peroxide
GGT: γ-glutamine transferase
HPA: Hypothalamic-pituitary-adrenal axis
Igs: Immunoglobulins
LDH: Lactate dehydrogenase
OT: Oxytocin
PDS: Postpartum dysgalactia syndrome
PMWS: Post-weaning multisystemic wasting syndrome
PRRS: Porcine reproductive and respiratory syndrome
sAA: Salivary alpha-amylase
sLip: Salivary lipase
TAC: Total antioxidant capacity
tADA: Total adenosine deaminase
TEA: Total esterase activity
TEAC: Trolox equivalent antioxidant capacity
